# Editor’s note

**DOI:** 10.1093/jac/dkab458

**Published:** 2022-02-07

Tabernero P, Swamidoss I, Mayxay M *et al*. A random survey of the prevalence of falsified and substandard antibiotics in the Lao PDR. *J Antimicrob Chemother* 2019; **74**: 2417–25. https://doi.org/10.1093/jac/dkz164.

This article published in the *Journal of Antimicrobial Chemotherapy* (*JAC*) has been retracted and republished^1^ to correct errors identified by the authors after publication of the original item.

Following publication of the original manuscript, the authors became aware that the reference ranges used to classify samples as meeting the pharmacopoeial specifications for assay [percentage of active pharmaceutical ingredient (%API) of the label claim] were incorrect.

The authors re-analysed the relevant data with the correct pharmacopoeial specifications for assay for the collected medicines and compared the results with those in the originally published manuscript.

The corrected version of the manuscript was reviewed by a member of *JAC*’s Editorial Board, the Editor-in-Chief and the original peer reviewer. Given the number and nature of the corrections required, the full corrected item has been republished in *JAC*.^1^

The authors apologize for their error, and the Journal apologizes for any confusion caused.
